# Computer-assisted textual analysis of free-text comments in the Swiss Cancer Patient Experiences (SCAPE) survey

**DOI:** 10.1186/s12913-020-05873-4

**Published:** 2020-11-10

**Authors:** Chantal Arditi, Diana Walther, Ingrid Gilles, Saphir Lesage, Anne-Claude Griesser, Christine Bienvenu, Manuela Eicher, Isabelle Peytremann-Bridevaux

**Affiliations:** 1 Department of Epidemiology and Health Systems, Center for Primary Care and Public Health (Unisanté), University of Lausanne, Route de la Corniche 10, 1010 Lausanne, Switzerland; 2 Medical Directorate, Lausanne University Hospital CHUV, rue du Bugnon 21, 1011 Lausanne, Switzerland; 3 Department of Policlinics, Center for Primary Care and Public Health (Unisanté), Rue du Bugnon 44, 1011 Lausanne, Switzerland; 4 Swiss Cancer Patient Experiences (SCAPE) survey steering committee, Lausanne, Switzerland; 5 Institute of Higher Education and Research in Healthcare (IUFRS), Route de la Corniche 10, 1010 Lausanne, Switzerland; 6 Department of Oncology, Lausanne University Hospital, Rue du Bugnon 21, 1011 Lausanne, Switzerland

**Keywords:** Cancer, Patient perspectives, Patient survey, Patient experiences, Quality of care, Textual analysis

## Abstract

**Background:**

Patient experience surveys are increasingly conducted in cancer care as they provide important results to consider in future development of cancer care and health policymaking. These surveys usually include closed-ended questions (patient-reported experience measures (PREMs)) and space for free-text comments, but published results are mostly based on PREMs. We aimed to identify the underlying themes of patients’ experiences as shared in their own words in the Swiss Cancer Patient Experiences (SCAPE) survey and compare these themes with those assessed with PREMs to investigate how the textual analysis of free-text comments contributes to the understanding of patients’ experiences of care.

**Methods:**

SCAPE is a multicenter cross-sectional survey that was conducted between October 2018 and March 2019 in French-speaking parts of Switzerland. Patients were invited to rate their care in 65 closed-ended questions (PREMs) and to add free-text comments regarding their cancer-related experiences at the end of the survey. We conducted computer-assisted textual analysis using the IRaMuTeQ software on the comments provided by 31% (*n* = 844) of SCAPE survey respondents (*n* = 2755).

**Results:**

We identified five main thematic classes, two of which consisting of a detailed description of ‘cancer care pathways’. The remaining three classes were related to ‘medical care’, ‘gratitude and praise’, and the way patients lived with cancer (‘cancer and me’). Further analysis of this last class showed that patients’ comments related to the following themes: ‘initial shock’, ‘loneliness’, ‘understanding and acceptance’, ‘cancer repercussions’, and ‘information and communication’. While **c**losed-ended questions related mainly to factual aspects of experiences of care, free-text comments related primarily to the personal and emotional experiences and consequences of having cancer and receiving care.

**Conclusions:**

A computer-assisted textual analysis of free-text in our patient survey allowed a time-efficient classification of free-text data that provided insights on the personal experience of living with cancer and additional information on patient experiences that had not been collected with the closed-ended questions, underlining the importance of offering space for comments. Such results can be useful to inform questionnaire development, provide feedback to professional teams, and guide patient-centered initiatives to improve the quality and safety of cancer care.

## Background

Health care research is becoming increasingly patient-centered, highlighting the importance of considering patients’ perspectives and experiences when evaluating the quality of care [1]. This has led to the development of patient-reported measures — reports that come directly from the patient about their health condition and experiences [2] — which provide the basis for a more holistic interpretation and assessment of care than traditional clinical outcome measures alone [3]. Patient-reported experience measures (PREMs) assess patients’ view on the delivery of care, such as communication with health care professionals and coordination of care [4, 5]. In contrast to satisfaction measures, experience measures focus on the underlying components of satisfaction by collecting information on what actually happened to patients during a hospital stay or a medical consultation [6]. In cancer care, specific experience measures have been advocated to account for the complex treatment pathways involved. Examples of cancer PREMs include those collected with the UK National Cancer Patient Experience Survey (CPES) and the US Consumer Assessment of Healthcare Providers and Systems (CAHPS) Cancer Care Survey [7, 8]. PREMs are usually collected through cross-sectional surveys using questionnaires with closed-ended questions producing quantitative data from a large sample of patients that can be used as indicators for the quality of health services. Questionnaires usually also include one or more open-ended questions, eliciting general comments (e.g. “Is there anything else you would like to tell us about your cancer care services?” [9, 10]) or more specific comments (e.g. “is there anything else you would like to tell us about your chemotherapy treatment?” [11]).

While quantitative results of closed-ended survey questions are widely published, analysis of free-text responses to open-ended questions or free-text sections are rarely performed and published. Indeed, manual thematic analysis of large amounts of text generated from open-ended questions remains time and resource intensive; therefore, the additional insights within this type of data often remain underutilized. However, such analyses can now be more easily performed thanks to information technology opportunities [12–15], yielding comparable results as manual qualitative analysis [16]. The analysis of free-text comments can serve various purposes: provide deeper insights on patient experiences, on specific closed-ended questions [17] or on subpopulations [18], identify issues that closed-ended questions might not reveal, guide the development of new survey questions and uncover issues with the survey or its methodology, and guide quality improvement initiatives [9–11, 17–23].

The aim of this study was to identify the underlying themes of patients’ experiences shared in their own words at the end of a cancer survey, using computer-assisted textual analysis, and to compare these themes with patient-centered care dimensions assessed in closed-ended questions (PREMs) to investigate how the textual analysis of free-text comments contributes to the understanding of patients’ experiences of care.

## Methods

### Study design

Textual analysis of free-text data collected between October 2018 and March 2019 in the multicenter cross-sectional Swiss Cancer Patient Experiences (SCAPE) survey.

### Population and setting

Patients aged 18 or older having received stationary or ambulatory care between January 1, 2018 and June 30, 2018 for breast, prostate, lung, colorectal, hematologic cancers (leukemia, lymphoma or myeloma) or melanoma, were eligible for inclusion in the SCAPE survey. The latter was conducted in four Swiss hospitals: the Lausanne university hospital, the Geneva university hospitals, and the cantonal hospitals of Fribourg and Valais.

### Measures

The SCAPE questionnaire, a comprehensive cancer care survey, was based on a French translation of the UK CPES [8] adapted to the Swiss context. It included 64 questions with a four- or five-point Likert-type scale response options on experiences of cancer care along the care pathway, from pre-diagnostic care to home care. The questions assessed the eight core dimensions of patient-centered care: information and education, coordination and integration of care, physical comfort, emotional support, respect for patients’ preferences, involvement of family and friends, continuity and transition, and access to care [24]. The questionnaire also included an overall rating of satisfaction with care (0 to 10 scale) and questions regarding demographic and socioeconomic characteristics as well as clinical and health status (age, sex, cancer type etc.). This amounted to a total of 94 multiple-choice questions and a final section for free-text comments (“If you wish to share cancer related experiences not covered in this questionnaire or if you have suggestions for improving cancer care, please share them on the following page”). Paper questionnaires were sent to the participants’ home address in October 2018; those returned by the end of March 2019 were considered for analysis.

### Textual analysis

To analyze the free-text comments, we performed a computer-assisted textual analysis using the IRaMuTeQ software (version 0.7 alpha 2, 2008–2014 Pierre Ratinaud). This is a tool particularly recommended for the analysis of large amounts of text [12]. Using the Reinert method [25], the software extracts recurrent themes using an algorithm that splits the text into segments which are then classified according to the co-occurrences of the words that compose these units. This classification results in a number of classes that are associated with typical vocabulary and typical extracts. The strength of association between the vocabulary and the classes is determined by Chi-square tests. Then, the researcher analyses the typical vocabulary and extracts for each class, and returns to the free-text comments to label and interpret the classes (first stage analysis). A repetition of the analysis can be performed on one or more of the classes, using the same method as described above (second stage analysis); the latter is typically done when one class contains a large amount and wide variety of vocabulary and themes. In addition to these analyses, the strength of association between modalities of latent variables (not used to build the classification variables) and the identified classes is determined with Chi-square tests, indicating if text included in the classes are specific to a modality of the latent variable. We included the following latent variables: age, sex, cancer type, overall satisfaction with care (approximate quartiles of a 0 to 10 scale: ≤7; 8; 9; 10), and the overall nature of each participant’s response (positive; negative; mixed; neutral), as coded by a researcher; unclear cases were discussed with one to two other researchers to reach consensus. All comments were considered for analysis except technical remarks concerning the survey.

### Data interpretation

Data interpretation was performed by three researchers who proceeded as follows: 1) they read all the comments while formatting the corpus to become familiar with the data; 2) once the software had classified the text into classes, they looked at the typical words and extracts associated within each class to make a preliminary thematic interpretation; 3) they went back to the original text in order to recontextualize the typical extracts and limit over-interpretation; 4) they discussed their thematic interpretation and labeling of classes with the multidisciplinary research team (i.e. researchers with backgrounds in nursing science, medical science, public health, and social science, and a cancer survivor who had taken part in the survey); and 5) they matched the thematic classes identified with the textual analysis to questions and dimensions of patient-centered care assessed in the closed-ended questions.

## Results

### Respondents’ characteristics

**Fig. 1:**
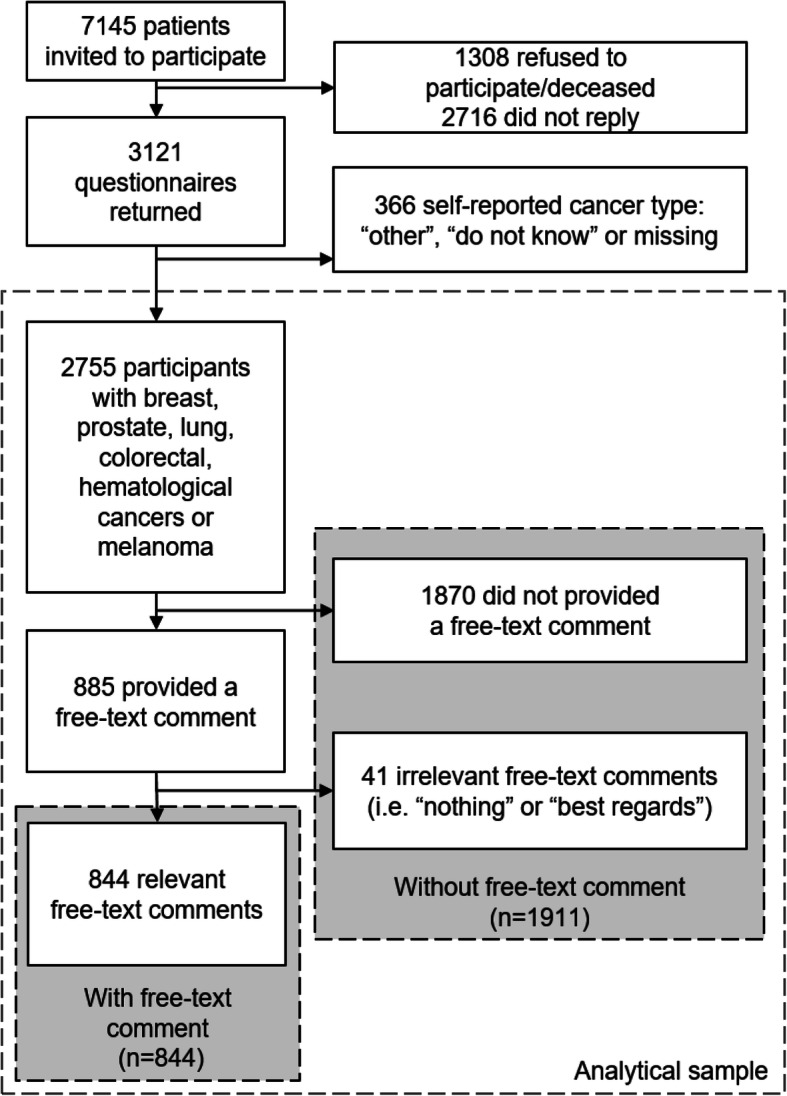
Flowchart of the SCAPE study

Of the 7145 individuals invited to participate in the survey, 3121 (43.7%) completed and returned the questionnaire. Of these, 2755 (88.3%) reported having either breast, prostate, lung, colorectal, hematologic cancers or melanoma and about a third wrote a comment at the end of the questionnaire (comment rate: 30.6%) and were included in the textual analysis (Fig. 1). As the ethics committee did not allow access to non-respondents’ personal data, comparisons between respondents and non-respondents were not possible. Mean age of those commenting was 62.1 years, 66.2% were women and 33.9% had completed tertiary education (Table 1). Participants who left a comment (*n* = 844) were more likely to be female, speak French, be more educated, and have breast cancer than those who did not leave a comment (*n* = 1912) (Table 1).

**Table 1:** SCAPE respondents’ characteristics, according to having left (or not) a free-text comment

Variable	With a free-text comment (*n* = 844) %	Without a free-text comment (*n* = 1911) %	Chi-squared *p*-value or Hedges’ g for means
Women	66.2	58.7	< 0.01
Age (mean)	62.1	64.7	0.20
French as principal language	90.2	83.9	< 0.01
Education			< 0.01
Primary	9.3	19.0	
Secondary	46.9	51.6	
Tertiary	43.8	29.4	
Overall satisfaction (0 to 10)			< 0.01
≤ 7	20.9	15.1	
8	24.5	27.2	
9	26.7	29.0	
10	28.0	28.7	
mean	8.4	8.6	0.13
Cancer type			< 0.01
Breast	44.9	38.0	
Prostate	7.1	9.3	
Lung	11.6	16.5	
Colorectal	9.7	10.8	
Hematologic	16.7	15.5	
Melanoma	5.2	5.2	
Several	4.7	4.6	
Hospital			0.34
Hospital 1	58.3	54.9	
Hospital 2	11.3	13.0	
Hospital 3	13.4	13.6	
Hospital 4	17.1	18.5	

### Characteristics and analysis of comments

**Fig. 2:**
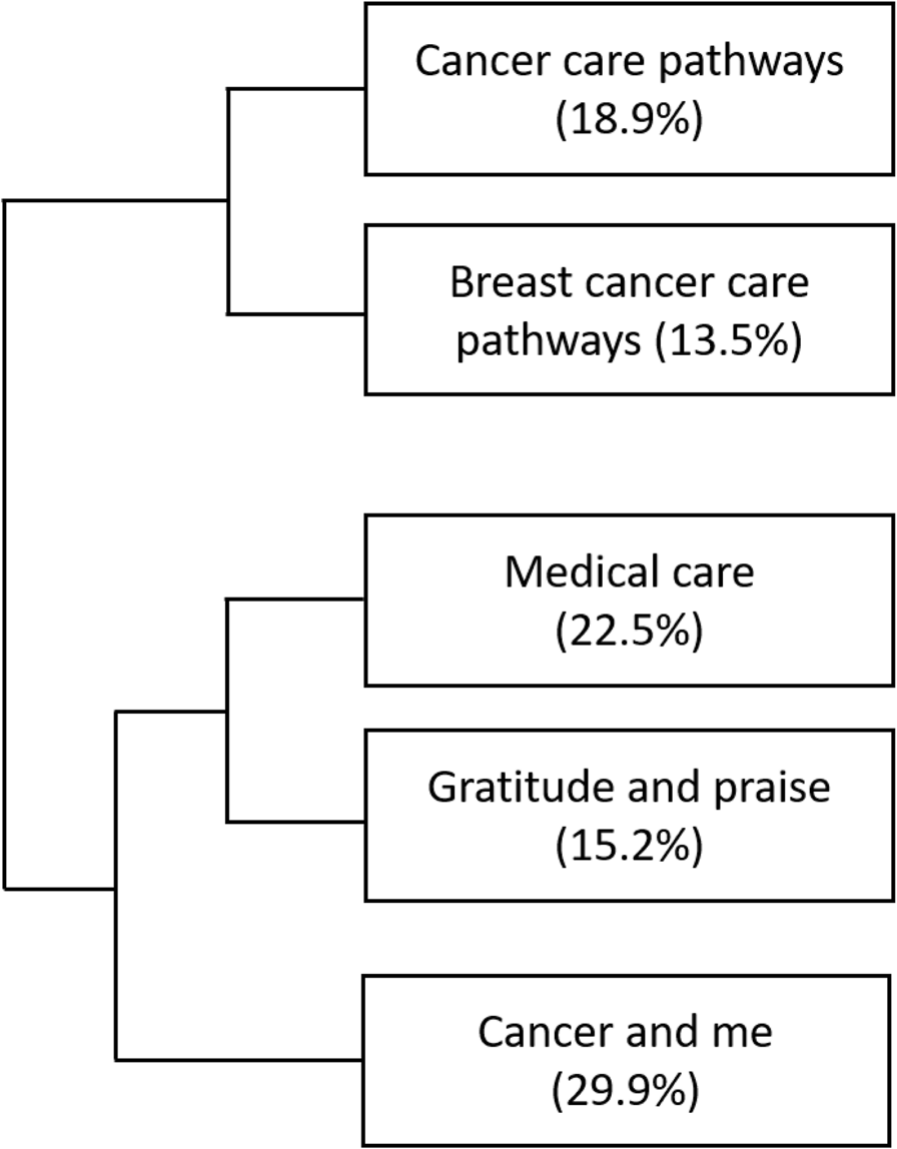
Hierarchical clustering dendrogram. (Dendrogram generated by IRaMuTeQ; percent of the text segments classified within each class indicated in parenthesis)

The 844 comments, ranging from one sentence to several pages of text, contained a total of 70′757 words; 28.0% of the comments were predominantly positive, 35.3% predominantly negative, 15.6% were an even mix of positive and negative statements and 21.1% were predominantly neutral. The software analysis allowed the classification of 95.2% of the text segments and identified five thematic classes. These classes were labelled by the research team as follows (percent of the text segments classified within each group indicated in parenthesis): ‘cancer care pathways’ (18.9%), ‘breast cancer care pathways’ (13.5%), ‘medical care’ (22.5%), ‘gratitude and praise’ (15.2%), and ‘cancer and me’ (29.9%). The structure of the classification is provided in a dendrogram showing the hierarchical clustering of the classes (Fig. 2), and details of the classes with typical words and extracts are provided in Table 2.

**Table 2:** Computer-assisted textual analysis, summary results

Class	Typical words	Typical excerpt	Associated modality (*p* < 0.05)
Cancer care pathways	Prostate, lung, leukemia, lymphoma, nodule, CT, to detect, operation, chemotherapy, radiotherapy, immunotherapy, months of the year, weeks, sessions	“My colon and liver cancer were discovered in July, operated in August, followed by chemotherapy from September to February”	Age ≥ 65 Men Prostate, lung and hematologic cancers Neutral comments Overall satisfaction 8/10
Breast cancer care pathways	Breast, tumor, cancer, malignant, metastasis, lymph nodes, gynecologist, screening, radiography mammography, echography, biopsy, to show/reveal, operate rate, removal	“hormone dependent breast cancer on the left side, discovered at a screening mammography […] lumpectomy followed by radiotherapy in January”	Age ≥ 75 Breast cancer Neutral comments Overall satisfaction 10/10
Medical care	Doctor (junior and senior doctors, oncologist, surgeons), medical department, patient, case, appointment, medical care, medical record, communication, organization, decisions, change, improve, follow, remind	“The post-operative care for the regular checkups is badly organized, the doctors change too often»	Women Negative and mixed comments Overall satisfaction ≤7/10
Gratitude and praise	Personnel, team, caregiver, nurse, oncology, thanks, gratitude, kindness, care, listening, competence, empathy, availability, quality, humanity, caring, extraordinary	“I’ll take this opportunity to thank all of the hospital personnel (nurses, doctors, radiologist, auxiliary staff, etc.) for their good care, their tact, their capacity to listen and their kindness”	Colorectal cancer Positive comments Overall satisfaction 10/10
Cancer and me	disease, side effects, pain, hormonotherapy, insurance, finances, information, understand, life, activity, psychologically, entourage, to help, think, feel, talk, face, live, search, find, suggest	“The disease destroyed my marriage and my family but especially the lack of support and psychological help in view of the situation that we had to face, we didn’t have enough information on the treatments, the side effects and financial help etc.”	Age < 65 yrs. Women Breast cancer Negative comments Overall satisfaction 8/10

#### ‘Cancer care pathways’

*Patient quotes (gender, age, type of cancer): “Lumpectomy, excision of the sentinel lymph node, 25 radiotherapy sessions at the [name of clinic], hormonotherapy” (Woman, 67, breast cancer); “Relapse in July 2016” (Woman, 71, hematologic cancer); “Currently, hormonotherapy and regular checkups” (Woman, 61, breast cancer).*

In this class, respondents recounted their cancer journey from diagnosis to treatment and follow up, mainly in a descriptive and neutral manner. Type and spread of cancer were often described, with details regarding cancer location, presence of cancer cells in lymph nodes as well as metastases. Respondents listed the examinations performed and types of treatment received. The course of disease, including remission and relapse, was also mentioned along with the frequency of follow-up appointments. Respondents often explained the timing and sequence of events. This class was overrepresented in those aged ≥65 yrs., in men, in those having left a neutral comment, with in those with overall satisfaction of 8/10, and respondents with prostate, lung and hematologic cancers.

#### ‘Breast cancer care pathways’

*Patient quotes: “It was during the mammography exam at the screening center in August 2017 that they discovered a 3 cm tumor” (Woman, 70, breast cancer); “12 lymph nodes are affected” (Woman, 49, breast cancer); “Checkups every 6 months”* (Woman, 66*, breast cancer*).

This class focused on breast cancer and was closely linked to the previous one. It included detailed descriptions of cancer care pathways, including technical terms, and specifications about the course of disease with temporal indications (diagnosis, treatment, follow-up, remission/relapse etc.). As in the previous class, respondents mentioned the examinations performed (mammography, echography, biopsy), details about the cancer location, size, type and spread, and the treatment received (mainly surgery). Again, the tone was rather neutral and descriptive. In addition, respondents in this class reported the reason for which they first sought medical care, such as a screening test or because they had noticed a lump in their breast. This class was overrepresented in respondents with breast cancer, in those aged ≥75 yrs., in those having left neutral comments, and with an overall satisfaction of 10/10.

#### ‘Medical care’

*Patient quotes: “The personnel were all stressed, including the doctors” (Man, 67, hematologic cancer); “Junior doctors change too often and don’t know our situation” (Woman, 55, breast cancer); “It took 26 phone calls to the [name of hospital] to get an appointment” (Woman, 72, breast cancer); “despite our requests, the records and test results do not always follow and the documents I receive are sometimes not up to date with the latest decision taken by the doctor” (Woman, 64, lung cancer).*

In this class, respondents related their experiences concerning cancer care, especially in regards to physicians. The majority of text in this class was negative. The frequent changes in staff at the junior physician level were often mentioned and described as problematic from the patient’s perspective. Staff were described as not being aware of the patients’ medical record, looking tired or overwhelmed and lacking time and supervision. The absence of senior physicians in direct patient care was also reported. Furthermore, respondents described poor organization and coordination of care including problems concerning their medical records, appointments and inter-professional communication. This class was overrepresented in women, in those having left predominantly negative or mixed comments, and in those with overall satisfaction of ≤7/10.

#### ‘Gratitude and praise’

*Patient quotes: “I’ll take this opportunity to thank all of the hospital personnel (nurses, doctors, radiologist, auxiliary staff, etc.) for their good care, their tact, their capacity to listen and their kindness” (Woman, 61, lung cancer); “The whole team saved my life, thank you from the bottom of my heart” (Man, 68, colorectal cancer).*

In this class, respondents expressed their gratitude and praise towards hospital staff as a whole or more specifically to professional groups or individuals including physicians, nurses, caregivers, therapists, technicians, social workers, receptionists and cleaning personnel. Respondents thanked health care professionals for the care provided, their competence, professionalism, kindness, empathy, benevolence, support and respect. The quality of care was highlighted both in terms of human relations as well as medical and technical expertise. Moreover, a few respondents mentioned the resulting effects including life-saving, reinforcing peace of mind, courage and trust. Those having left positive comments, those with an overall satisfaction of 10/10 and respondents with colorectal cancer were overrepresented in this class.

#### ‘Cancer and me’

**Fig. 3:**
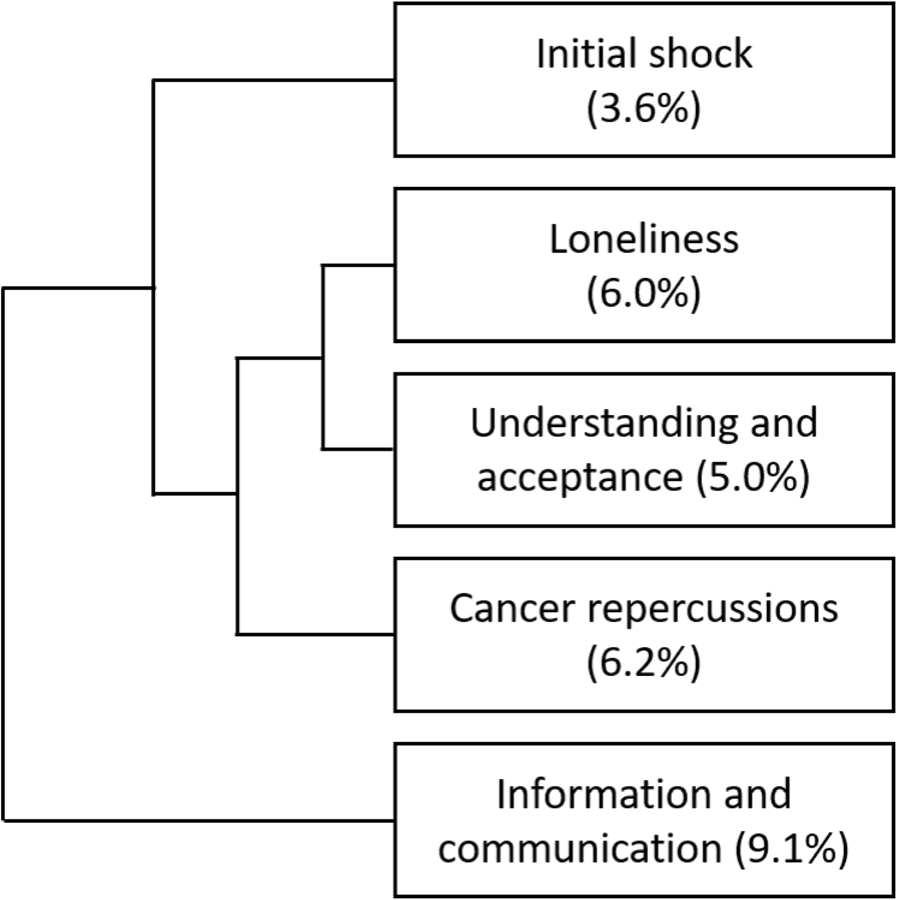
Hierarchical clustering dendrogram of the ‘cancer and me’ class. (Dendrogram generated by IRaMuTeQ; percent of the text segments classified within each class indicated in parenthesis)

As the ‘cancer and me’ class included a large amount of text (29.9%), we repeated the analyses on this class separately in order to have a more detailed understanding of its content. The secondary analysis of this class resulted in five sub-classes, labelled as follows (percent of text segments classified within each group indicated in parenthesis): ‘initial shock’ (3.6%), ‘loneliness’ (6.0%), ‘understanding and acceptance’ (5.0%), ‘broad impacts of cancer’ (6.2%), and ‘information needs’ (9.1%). The structure of the ‘cancer and me’ sub-classification is provided in a dendrogram showing the hierarchical clustering of the subclasses (Fig. 3).

##### ‘Initial shock’

*Patient quotes: “I was completely under shock and had to return by car alone; the shock came from the brutal communication of the diagnosis” (Woman, 56, lung cancer); “There is no good way to announce cancer, once the word is said it’s a real tsunami” (Woman, 61, breast cancer); “At the time of the cancer diagnosis, under shock, we don’t retain anything, the need for information comes later” (Woman, 52, colorectal cancer).*

In this class, respondents reported negative experiences regarding the manner and circumstances in which they were told about the cancer diagnosis or the treatment needed, resulting in a state of shock. Respondents’ criticism included being told the diagnosis in a brusque manner, without empathy, over the phone, in a shared hospital room with little privacy, or when alone without the presence of a friend or family member. The sense of shock was also reported by patients who reported being told in a tactful manner, resulting in difficulty in retaining information and in making decisions at the time. Suggestions for improvement included receiving written information on the disease, disease course and treatment after receiving the news, in a follow-up appointment. This class was overrepresented in respondents having left negative comments.

##### ‘Loneliness’

*Patient quotes: “I struggle alone to pull through” (Woman, 54, breast cancer); “We feel alone and not always heard. At each appointment with the oncologist we repeat our side effects, they are recorded in the computer as if it were normal” (Woman, 51, breast cancer); “And then comes the hormonotherapy, no more information, just a prescription and ‘we’ll see each other in 3 months’ … we discover the side effects alone, they can be very difficult. We just know that it will be for a long time, a very long time.” (Woman, 53, breast cancer); “I felt alone, abandoned by the doctors, lost after the treatment” (Woman, 42, breast cancer).*

In this class, respondents described the loneliness felt during treatment, either lacking support from medical personnel or from friends and family. Loneliness was also expressed regarding the side effects of treatment, especially when met with a lack of understanding or solutions from medical staff. Respondents expressed how they had to deal with these side effects “alone”, turning for example to alternative medicines. Another recurrent theme was the loneliness felt at the end of treatment, when respondents felt “abandoned” and having to deal by themselves with the difficulties of returning to work or with the incapacity to work, resulting in financial difficulties for some respondents. This class was overrepresented in respondents with an overall satisfaction of 9/10, those having left positive comments and with prostate cancer.

##### ‘Understanding and acceptance’

*Patient quotes:* ***“****I wanted to function like before my illness, everyone was pushing me, they didn’t understand, didn’t accept that I’m unable to” (Woman, age unknown, breast cancer); “The empathy, interest, understanding, asking for news and the messages of support from family, friends, work colleagues and acquaintances are very important in order to accept the disease and always useful for the morale” (Man, 54, melanoma).*

Elements concerning the patients’ understanding and acceptance of the disease and its treatment were expressed in this class. On the one hand, the lack of information and poor communication with medical staff or the lack of opportunities to share experiences with fellow patients created barriers to understanding and acceptance of the disease. On the other hand, adequate information from staff or external sources (e.g. websites, books) facilitated understanding and acceptance. “Understanding” from others was also expressed as either present and of great importance to patients, or as absent. Lack of understanding, be it from medical staff, loved ones, employers, colleagues or insurance companies, was a source of suffering for some patients. This class was overrepresented in respondents with hematologic cancer and in those with an overall satisfaction of 10/10.

##### ‘Cancer repercussions’

*Patient quotes: “How to live a normal life with this relentless pain” (Man, 54, hematologic cancer); “The disease destroyed my marriage and my family” (Woman, 35, breast cancer); “After 6 months of sick leave and three surgical interventions my employer pushed me to leave” (Woman, 54, breast cancer).*

In this class, the respondents’ discourse focused on the period during which the treatment took place and the way in which the disease and its treatment disrupted their quality of life as it affected their physical and emotional health. They described the negative impacts on different aspects of their life including friends, family, work and leisure. For example, respondents reported the difficulties they had to reconcile their professional life with care (e.g. amount of appointments, work interruptions). Respondents also mentioned adverse effects of treatments, such as pain or insomnia, as well as financial issues linked with treatment costs and their inability to work. From within this turmoil, respondents related their aspirations to be able to lead once again – a “normal life”. No category of respondents was overrepresented in this class.

##### ‘Information and communication’

*Patient quotes: “I rarely received the essential information spontaneously; [it was a] wrestling match to obtain answers to my questions” (Man, 66, lung cancer); “It’s necessary to improve information to patients about side effects, which are sometimes harder than the cancer itself” (Man, 60, colorectal cancer); “Nurses are more open than the doctors to talking about benefits of homeopathy, osteopathy and hypnosis. It’s a complementarity that helped me a lot because I decided to do something that made me feel good rather than to “suffer” under the prescribed treatments” (Woman, 55, breast cancer).*

This class includes comments on the information and communication around cancer. Respondents expressed difficulties obtaining information on the disease, the treatment, the side effects, their results and medical reports, as well as on the range of programs and help available to patients. Shortcomings included the content, the amount (too much/too little), the form (oral/written) as well as the timing (too early/too late) of the information delivered. Respondents also provided suggestions to improve communication; for example, they proposed that information could be communicated orally and in written form using clear simple language. A particularly frequent theme in this class was the lack of information on side effects and sequelae, the lack of understanding, compassion and help for side effects from medical professionals and the desire for more information on complementary therapies. Medical professionals, especially physicians, were described as being closed to complementary therapies, though they featured regularly as a means by which patients coped with the side effects. No category of respondents was overrepresented in this class.

### Comparisons with patient-centered dimensions from closed-ended questions

Comparisons between the thematic classes from the textual analysis of free-text comments and the patient-centered care dimensions assessed in the closed-ended questions are presented in Table 3. In general, the themes that emerged from free-text comments were related to the patient-centered care dimensions and other components assessed in closed-ended questions, but went beyond the evaluation of whether an episode of patient-centered care happened or not. Indeed, the thematic classes provided more details on specific episodes of both positive and negative experiences of care and on qualities of healthcare professionals not fully assessed by the closed-ended question. While most closed-ended questions related to experiences of care within the last 12 months, free-text comments included experiences that happened during a larger period. In addition, the content of the thematic classes also related to the personal experience of receiving the diagnosis and living with cancer, as well as the impacts of poor experiences of cancer care. These aspects were not directly evaluated by PREMs.

**Table 3:** Comparisons and contributions of thematic classes to patient-centered care dimensions

Thematic classes of free-text comments	Patient-centered care dimensions of closed-ended questions ***Exemples of questions***	Contributions of thematic analysis of free-text comments to closed-ended questions
Cancer care pathways and breast cancer care pathways	Clinical information *What is your principal cancer diagnosis?* *How long has it been since you were first treated for this cancer?* *What type(s) of treatment have you received?*	Thematic analysis added details on the temporal course of cancer diagnosis, care and clinical pathways. Note: the questions related to clinical information were not part of the patient-centered care dimensions assessed by the closed-ended questions.
Medical care	Coordination and integration of care *Did the different people treating and caring for you work well together to give you the best possible care?* *In your opinion, were there enough nurses on duty to care for you in hospital?*	Thematic analysis added details on specific issues related to coordination between different healthcare professionals and integration of services: e.g. negative comments on individual physicians and specific episodes, comments on aspects such as ‘staff changes’ or ‘doctor looking tired or overwhelmed’ not evaluated by closed-ended questions.
Gratitude and praise	Emotional support *Did you trust the doctors/nurses treating you?* *During your hospital visit, did you find someone on the hospital staff to talk to about your worries and fears?*	Thematic analysis added detailed descriptions of and reasons for the positive aspects of care and relationships with a wide variety of actors (e.g. doctor, nurse, therapist, social worker, receptionist, cleaning personnel): specific thanks, gratitude, and qualities (e.g. kind, caring, competent, empathic, attentive, available, extraordinary, dedicated, excellent).
Cancer and me: initial shock	Respect for patients’ preferences *How do you feel about the way you were told you had cancer? [done sensitively?]* Information and education *Did you understand the explanations about what was wrong with you?*	Thematic analysis added description of the impact of learning they had cancer (e.g. shock), which was not assessed in closed-ended questions. Patients’ needs and suggestions to improve the delivery of the diagnosis and other important medical information were also a valuable contribution obtained from thematic analysis.
Cancer and me: loneliness	Emotional support *During your hospital visit/while you were being treated as an outpatient, did you find someone on the hospital staff to talk to about your worries and fears?* Continuity and transition *Did hospital staff give you information about support or self-help groups for people with cancer?* *During/after your cancer treatment, did you receive enough care and support from health/social services?*	Thematic analysis added description of the impact of poor experiences of care regarding emotional support and continuity of care. This included: patients’ feelings of loneliness during and after treatment (e.g. while managing side effects, accessing complementary medicine, or resuming a professional activity after treatments); and patient’s needs and suggestions on how to alleviate loneliness (e.g. help & support with administration & finances).
Cancer and me: understanding and acceptance	Information and education *Did you understand the explanations about what was wrong with you?* *Were the results of the diagnostic test explained in a comprehensible manner?* Involvement of family and friends *When you were first told that you had cancer, had you been told you could bring a family member or friend with you?*	Thematic analysis added information on support (or lack thereof) from families, friends or support groups in accepting the disease, and on the negative impact of lack of understanding from medical staff, employers, and insurance companies.
Cancer and me: cancer repercussions	Health-related quality of life *I have a lack of energy; I am able to enjoy life; I worry that condition will get worse; I have nausea; I am content with the quality of life right now; I am sleeping well; I have pain [‘not at all’ to ‘very much’]* Continuity and transition *Did hospital staff discuss with you or give you information about the impact cancer could have on your day to day activities?* *Did hospital staff give you information about how to get financial help or any benefits you might be entitled to?* Physical comfort *Do you think the hospital did everything they could to help control you pain?*	Thematic analysis added detailed descriptions of how cancer affected patients’ (and their families’) quality of life, including physical and emotional health, family, social and professional life, financial impact, and fears, as well as reports on how difficult it is to have a “normal life” after cancer treatment. Note: the seven questions on health-related quality of life were not part of the patient-centered care dimensions assessed by the closed-ended questions; they were from the rapid version of the functional assessment of cancer therapy-general (FACT-G7) instrument.
Cancer and me: information and communication	Information and education *Were the possible side effects of treatment(s) explained in a comprehensible manner?* Continuity and transition *Were you offered practical advice and support in dealing with the side effects of your treatment(s)?* Coordination and integration of care *Did the different people treating and caring for you work well together to give you the best possible care?* Complementary medicine *Have you used any complementary medicine?*	Thematic analysis added information on patients’ needs and suggestions for: better access to and explanation regarding medical information (including side-effects); better communication between healthcare professionals; and better recognition and integration of complementary medicine by doctors

## Discussion

This computer-assisted textual analysis of free-text comments provided by patients with cancer in response to an invitation to share cancer-related experiences and suggestions for improving care at the end of the SCAPE questionnaire (31% comment rate) allowed us to identify five main thematic classes: ‘cancer care pathways’, ‘breast cancer care pathways’, ‘medical care’, ‘gratitude and praise’, and finally ‘cancer and me’. This latter class was further divided into five subclasses: ‘initial shock’, ‘loneliness’, ‘understanding and acceptance’, ‘cancer repercussions’, and ‘information and communication’. Apart from the first two classes, the thematic classes of the free-text comments related more to the personal and emotional experience and consequence of having cancer and receiving care, while the dimensions of the closed-ended questions assessed mainly the factual aspects of people’s experience of patient-centered care.

We also observed a sharp contrast within the free-text comments between the factual descriptions of medical history and care pathways, grouped in the ‘cancer care pathways’ and ‘breast cancer care pathways’ themes and the more personal aspects of living with cancer, especially present in the ‘cancer and me’ class. The described care pathways encompassed diagnosis to treatment through to remission or relapse, framed by key examinations, interventions, treatments and results. On the other hand, personal elements included emotional and social aspects related to cancer and care, such as coping with cancer and its treatments and the interactions with health care providers. This distinction in the free-text comments may reflect the way patients and health professionals interact. Their interaction has been described as being structured by two distinctive needs: professionals’ needs of ‘knowing and understanding’ and patients’ needs of ‘feeling known and understood’ [26]. In our results, the former would be expressed in the neutral description of the care pathways whereas the latter would be expressed through the more personal and embodied experiences. The ‘cancer and me’ class is a typical example of the participants’ need of ‘feeling known and understood’ with particularly rich illustrations of the vast array of challenges faced by respondents. Confronted with cancer, patients have to absorb the initial shock of diagnosis, deal with feelings of loneliness, seek adequate information, understand and accept the disease, and manage the repercussions of cancer on their lives. This class included comments related to how cancer and cancer symptoms had an impact on the person, on *self*, on the emotional experiences of living and coping with cancer, aligning with experiences reported in previous meta-analyses on living with cancer and symptom experiences [27, 28]. Compared with the closed-ended questions on emotional support, the ‘cancer and me’ class went beyond positive or negative experiences to encompass descriptions of living with cancer.

The challenges patients face when confronted with cancer and cancer symptoms, as well as the discontinuity of care brought about by the frequent changes at the junior physician level along with the faulty inter-professional communication expressed in the ‘medical care’ theme, illustrates the need for ongoing and better care coordination and multifaceted support. This theme is a recurrent theme reported in other similar studies on the analysis of free-text comments, highlighting the importance of this aspect to individuals with cancer [9, 11, 19, 21]. The importance of this theme for patient-centered care is also reflected in the questionnaire as the ‘coordination and integration of care’ dimension was assessed in six closed-ended questions. Recent systematic reviews have shown that cancer care coordination interventions, such as increased communication across multidisciplinary teams, patient navigation, home telehealth, self-management education and nurse case management, have the potential to improve a range of cancer related outcomes, including measures of patient experience with care, and quality of life [29–32]. It may be beneficial to further develop such interventions.

In all classes, except the two classes about care pathways, participants expressed positive and negative statements when describing their care experiences, which may further guide quality improvement initiatives. Negative comments identify aspects that need change while positive comments help staff identify how they are valued and what they are doing well, providing motivation to continue [33]. The negative feedbacks and suggestions provided by the study respondents mostly concerned the four following areas: 1) the frequent staff changes at the junior physician level; 2) communication issues, with particular emphasis on the lack of information (oral and written) on side-effects and complementary therapies; 3) the manner and circumstances in which the cancer diagnosis and treatment were communicated; and 4) the lack of support from health care professionals throughout the treatment process, with particular shortcomings in regards to side effects and end of treatment. The first two areas of care, i.e. ‘staff changes’ and ‘communication about complementary therapies’, were not specifically assessed with closed-ended questions, thus providing useful information for questionnaire improvement. On the other hand, the survey contained several closed-ended questions on the communication of diagnosis as well as information and support about side-effects. Quantitative results of these questions also showed more negative experiences compared to questions on other dimensions. Thus, these latter themes from free-text comments consolidated the quantitative findings and provided in-depths illustrations of these issues and concerns around communication and support, which have also been reported in other studies [19, 21].

Concerning positive feedback, an entire class was dedicated to expressing ‘gratitude and praise’ toward health care professionals for their medical/technical expertise and the quality of human relations. If respondents felt the need to express gratitude and difficulties in this questionnaire, it maybe that they did not have sufficient opportunities to do so during care and in the section of the questionnaire with closed-ended questions. Previous studies have also reported frequent positive feedback in free-text comments, ranging from one third of comments related to appreciation and gratitude [9] to two thirds of comments being positive [19]. These comments have been very useful when communicating the results to the participating hospitals in our study, and could also be used to inform a “safety II culture” approach, which builds on ‘things that go right’ instead of focusing primarily on critical incidents and adverse events [34].

The use of a computer-assisted textual analysis method represents the main strength of this study, having allowed comprehensive analysis of all free-text comments within a large survey sample in a time-efficient way. It resulted in the identification of important themes for individuals with cancer, some of which have been reported in other studies using different approaches, both inductive and deductive and manual or computer-based methods [9, 11, 19, 21]. The results of this study need nevertheless to be interpreted taking into consideration the following limitations. First, our results reflect experiences of patients who transited within one of the four hospitals involved in recruitment. However, this includes both university and non-university hospitals covering a wide range of French-speaking Switzerland. Secondly, since the questionnaire was only available in French, patients not speaking the language well would not have been able to express their experiences fully and may experience care differently [35]. Thirdly, free-text comments were written by a third of the respondents, who were more likely to be female, speak French, be more educated, and have breast or lung cancer. This may bias results, though mean age and overall satisfaction with care between those having left and those not having left a comment were similar. Finally, a frequent criticism made about computer-assisted text analyses is that the results are not sensitive to the context because the text is fragmented into analytic units. The impact of this criticism has however been limited, as the researchers frequently returned to the original text to consider the context and validated their interpretation with a patient representative.

## Conclusions

In cancer patient experience surveys, providing space for free-text allows respondents to express themselves in their own words and to report in more details about their personal experiences of living with cancer, contributing to the better understanding of their experiences and going beyond the standardized evaluation of patient-centered care. Indeed, the analysis of free-text comments sheds light on important themes and aspects of care that patients choose to report freely and that closed-ended questions may not reveal, providing complementary insights. It also underlines the importance of offering space for comments and highlights the diversity of cancer patients’ journeys and experiences, encompassing aspects outside of health care. Such results are particularly useful to inform questionnaire development, provide feedback to hospitals and healthcare teams, and guide quality and safety of care initiatives aiming at enhancing the patient-centeredness of care and improving the cancer care experience overall.

## Data Availability

The French version of the survey is available upon request. The data that support the findings of this study are available on request from the corresponding author [CA]. The free-text data are not publicly available due to them containing information that could compromise research participant privacy.

## Abbreviations

CPES: Cancer Patient Experience Survey
CAHPS: Consumer Assessment of Healthcare Providers and Systems
IRaMuTeQ: Interface de R pour les Analyses Multidimensionnelles de Textes et de Questionnaires
PREM: Patient-reported experience measure
SCAPE: Swiss Cancer Patient Experiences
